# Collagen triple helix repeat containing-1 negatively regulated by microRNA-30c promotes cell proliferation and metastasis and indicates poor prognosis in breast cancer

**DOI:** 10.1186/s13046-017-0564-7

**Published:** 2017-07-12

**Authors:** Yuan-hui Lai, Jian Chen, Xiao-ping Wang, Yan-qing Wu, Hai-tao Peng, Xiao-hong Lin, Wen-jian Wang

**Affiliations:** 1 Department of Thyroid and Breast Surgery, the First Affiliated Hospital of Sun Yat-sen University, Guangzhou, Guangdong 510700 China; 2 Department of Organ Transplantation, the First Affiliated Hospital of Sun Yat-sen University, Guangzhou, Guangdong 510700 China; 3 Guangzhou First People’s Hospital, Guangzhou Medical University, Guangzhou, Guangdong 510180 China; 4 Laboratory of Department of Surgery, the First Affiliated Hospital, Sun Yat-sen University, 58 Zhongshan Road II, Guangzhou, Guangdong 510080 China

**Keywords:** Breast cancer, Prognosis, Metastasis, CTHRC1, miR-30c

## Abstract

**Background:**

Collagen triple helix repeat containing-1 (CTHRC1), which was firstly identified overexpressed in the adventitia and neointima of injured rat arteries, could inhibit collagen expression and increase cell migration. It was then found to be ubiquitously expressed in numerous cell types such as fibroblasts and smooth muscle cells, and aberrantly up-regulated in several malignant tumors. However, the functional role of CTHRC1 and its related mechanism in breast cancer still remains unclear.

**Methods:**

CTHRC1 expressions in breast cancer tissues and cells were assessed by qRT-PCR, western blot and immunohistochemistry. The relative expression level of miR-134, miR-155, miR-30c and miR-630 in breast cancer cells respectively was detected by qRT-PCR. Wild type (Wt) and Mutant type (Mut) CTHRC1 3’UTR sequences were cloned into a psi-CHECK2 reporter vector, and the relative luciferase activity was detected by dual-luciferase reporter assay in indicated cells. The effect of ectopic expression of miR-30c or gain and loss of CTHRC1 on cell viability, cell proliferation, cell cycle progression and apoptosis, cell invasion and migration was respectively detected by CCK-8 assay, colony formation assay, flow cytometry analysis, transwell invasion/migration assay. Protein levels of β-catenin, active β-catenin, normal and phosphorylated form of GSK-3β were detected by western blot in indicated cells. Immunofluorescence staining of β-catenin was performed to observe nuclear localization.

**Results:**

We found CTHRC1 was frequently up-regulated in human breast cancer cells and tissues. Then our cohort study and further meta-analysis validated high expression of CTHRC1 was associated with aggressive clinicopathological features and poor clinical outcome of breast cancer patients. In addition, CTHRC1 promoted cell proliferation, invasion and migration and suppressed cell apoptosis in breast cancer, which might be by activating GSK-3β/β-catenin signaling and inhibiting Bax/Caspase-9/Caspase-3 signaling respectively; and these biological functions of CTHRC1 could be directly negatively regulated by miR-30c.

**Conclusion:**

Taken together, we identified the role of miR-30c/CTHRC1 axis in breast cancer progression and demonstrated CTHRC1 may serve as a prognostic biomarker and therapeutic target for breast cancer.

**Electronic supplementary material:**

The online version of this article (doi:10.1186/s13046-017-0564-7) contains supplementary material, which is available to authorized users.

## Background

Breast cancer is the most frequently diagnosed cancer and the leading cause of cancer death among females worldwide [1]. Despite the improvement in early clinical detection and therapy strategies of breast cancer, the prognosis of breast cancer patients still remains unsatisfying [2]. Therefore, it is necessary to understand the mechanisms that underlie breast cancer progression and thus identify new precise therapeutic strategies.

Collagen triple helix repeat containing-1 (CTHRC1) is a secreted 28-kDa protein, which was firstly identified overexpressed in the adventitia and neointima of injured rat arteries, could inhibit collagen expression and increase cell migration [3–5]. CTHRC1 was then found to be ubiquitously expressed in numerous cell types such as fibroblasts and smooth muscle cells, and aberrantly up-regulated in several malignant tumors, including melanoma, and cancers of gastrointestinal tract, liver, lung, ovary and so on [6–8]. Yamamoto et al. [9] have documented that cell surface anchored CTHRC1 can stabilize the physical interaction between Wnt ligands and Frizzled receptors, and selectively activate the planar cell polarity pathway of Wnt signaling to regulate cell motility and taxis. In our previous studies, we have found that CTHRC1 promotes non-small cell lung cancer (NSCLC) cell aggressiveness by targeting Wnt/β-catenin pathway, and may serve as a novel biomarker for NSCLC patients [7]. Currently, there is little information about the role of CTHRC1 in breast cancer progression. Kharaishvili et al. [10] performed immunohistochemical study of CTHRC1 in breast cancer with parallel analysis of periostin and versican and found that combined evaluation of stromal expression of CTHRC1 and periostin could serve as a potential marker for breast cancer bone metastasis prediction. Later Kim et al. [11] found CTHRC1 over-expression was significantly associated with clinicopathological factors of poor prognosis in invasive ductal carcinoma. However, the functional role of CTHRC1 and its mechanism in breast cancer still remains unclear.

In the present study, we demonstrated that CTHRC1, negatively regulated by miR-30c, could promote breast cancer cell proliferation, invasion and migration and suppress cell apoptosis. In addition, our cohort study and further meta-analysis corroborated high expression of CTHRC1 in primary tumors of breast cancer patients is associated with poor clinical outcome.

## Methods

### Patients and tissue samples

Primary invasive ductal carcinomas of breast were obtained from 121 female patients at the Department of Breast and Thyroid Surgery, the First Affiliated Hospital, Sun Yat-sen University, from January 2007 to December 2012. Pathological diagnosis, as well as ER, PR and Her2 status, was verified by two different pathologists. Patients with invasive carcinomas, other than DCIS, underwent six cycles of postoperative adjuvant chemotherapy with FAC regimen (5-fluorouracil 500 mg/m^2^, doxorubicin 50 mg/m^2^, and cyclophosphamide 500 mg/m^2^). Subsequently, patients with ER(+) tumors underwent endocrine therapy according to NCCN guideline. No distal metastasis was identified in the patients upon diagnosis. In addition, fresh samples of normal breast tissue, benign breast tumor tissues and invasive ductal carcinoma tissues were collected from patients who had undergone mastectomy or lumpectomy for benign or malignant breast diseases. All samples were snap-frozen for mRNA assessment and were collected with informed written consent from patients. The complete clinical and pathological features of these patients were collected and stored in our database by a researcher fellow. The study protocol followed the Ethical Guidelines of the 1975 Declaration of Helsinki, revised in 2000. All related procedures were performed with the approval of the Internal Review and the Ethics Boards of the First Affiliated Hospital, Sun Yat-sen University. All research protocols strictly complied with REMARK guidelines for reporting prognostic biomarkers in cancer [12].

### Immunohistochemistry

Archived paraffin-embedded tumor tissues collected from 121 consecutive patients with breast cancer treated in our hospital between 2007 and 2012 were used for tissue microarray construction and immunohistochemistry (IHC). The IHC was performed using the polymer HRP detection system (Zhongshan Goldenbridge Biotechnology, Beijing, China) and antibodies were listed in the Additional file 1: Table S3. CTHRC1 expression level was scored semi-quantitatively using the IRS (immunoreactive score) = SI (staining intensity) × PP (percentage of positive cells) as described [13, 14]. Briefly, SI was determined as 0, negative; 1, weak; 2, moderate and 3, strong. PP was defined as 0, <1%; 1, 1–10%; 2, 11–50%; 3, 51–80% and 4, >80% positive cells. Five visual fields from different areas of each tumor were used for the IRS evaluation. IRS ≤ 4 was defined as low CTHRC1 expression and IRS > 4 were defined as high CTHRC1 expression.

### Cell lines and cell culture

In this study, human breast cancer cell lines, SK-BR-3, MDA-MB-231, MDA-MB-468, MCF-7, and BT549 as well as MCF-10A breast epithelial line were obtained from the American Type Culture Collection (ATCC, Manassas, VA). The cells’ characters were as described by Kao et al. [15]. These cells were cultured according to the recommended protocols.

### RNA extraction and real-time quantitative polymerase chain reaction (qRT-PCR)

TRIzol^®^ Reagent (Life Technologies, Carlsbad, CA) was used to isolate total RNA from frozen patient samples and cell lines according to the manufacturer’s protocol. cDNA was synthesized using the universal cDNA synthesis kit (Toyobo, tokyo, JP). The RNA was then reverse-transcribed to obtain cDNA by the universal cDNA synthesis kit (Toyobo, tokyo, JP) at 37 °C for 50 min. The cDNA was subjected to quantitative real-time PCR (qRT-PCR) using the SYBR Green PCR Kit (Roche Life Sciences, Switzerland) and the assay was performed on an PRISM 7300 Sequence Detection System (Applied Biosystems, CA). 18SrRNA was used as an internal control for CTHRC1 and U6 for microRNAs. The relative levels of expression were quantified and analyzed. The experiments were done in triplicate. The primers were all synthesized and bought from Sangon Biotech (Shanghai, China). The detailed procedure was as Gong et al. described [16]. The primer sequences are listed in the Additional file 1: Table S2.

### Western blot

Total proteins were extracted with RIPA lysis buffer and separated by SDS-PAGE and then transferred to the PVDF membrane (Roche Life Sciences, Switzerland). The membrane were blocked with 5% skimmed milk and incubated with the appropriate antibody. The antigen-antibody complex on the membrane was detected with enhanced chemiluminescence regents (Thermo Scientific, Waltham, MA). The antibodies are listed in the Additional file 1: Table S3.

### Immunofluorescence for cells

Cells grow at glass coverslips, and then were fixed with 4% paraformaldehyde in phosphate-buffered saline (PBS) with 0.2% Triton. Cells were then blocked for an hour with 1% bovine serum albumin (BSA) followed by incubation with primary antibody overnight at 4 °C. Cells were washed and incubated with appropriate secondary antibody and DAPI. The slides were imaged using an inverted fluorescence microscope DMI4000B (Leica, Wetzlar, Germany). The antibodies and reagents are listed in the Additional file 1: Tables S3 and S4.

### Transfection

In this study, BT549 and HEK293T cells were transfected with miR-30c mimics, negative control, miR-30c inhibitor and inhibitor negative control. CTHRC1 expression vector without 3′-UTR was constructed by inserting its CDS sequence into the psiCHECK2 vector (Promega, USA). qRT-PCR analysis were performed to confirm the transfection efficiency (Additional file 2: Figure S3A). For siRNA and stable plasmid DNA transfection, cells were transfected into BT549 cells with specific siRNA duplexes targeting CTHRC1 or pcDNA3 vector expressing CTHRC1 construct using Lipofectamine 2000 (Invitrogen) according to the manufacturer’s instruction. The overexpression level of CTHRC1 and silencing efficiency of each siRNA was assessed by qRT-PCR. Gain of CTHRC1 resulted in significant increase in its expression (Additional file 2: Figure S3B). CTHRC1-siRNA-2 caused the lowest level of CTHRC1 and then was selected for further study (Additional file 2: Figure S3C). The overexpression and knock-down level of CTHRC1 was further validated by western blot (Additional file 2: Figure S3D).

The above used sequences are listed in the Additional file 1: Table S5.

### In vitro cell behavior assays

To assess the cell viability, the Cell Counting Kit-8 (CCK-8) (Dojindo Molecular Technologies, USA) was used according to the manufacturer’s instructions. After transfection for 24 h, cells were seeded in 96-well plates. The cell viability was detected by adding WST-8 at a final concentration of 10%, and the absorbance of these samples were measured at 450 nm on a Microplate reader (Molecular Device, SpectraMax M5e, USA) every 24 h for 4 days. The data derived from triplicate samples are presented as mean ± SD.

For colony formation assay, cells were seeded into 6-well plates at a density of 500 cells/well and cultured for 2 weeks at 37 °C. The numbers of colonies per dish were counted after staining with crystal violet (Beyotime Institute of Biotechnology). Only positive colonies (diameter > 40 um) in the dishes were counted and compared.

Cell cycle analysis was conducted by flow cytometry (Beckman-Coulter, Fullerton, CA, USA) using a Propidium Iodide (PI) cell cycle detection kit (Beyotime Institute of Biotechnology, Beijing, China). Cell apoptosis analysis was conducted by flow cytometry (Beckman-Coulter, Fullerton, CA, USA) using Annexin V-FITC/PI Apoptosis Detection Kit (Keygen Biotechnology, Nanjing, China).

For the transwell invasion assay, 1 × 10^5^ cells in serum-free medium were seeded into the upper chamber of 8-μm transwell inserts with a matrigel coated membrane (BD Biosciences, Franklin Lakes, NJ), while medium containing 10% bovine serum albumin was in the lower chamber. After several hours of incubation at 37 °C, gel and cells in the upper chamber were removed carefully and cells adhering to the underside of the membrane were stained with 0.1% crystal violet (Beyotime Institute of Biotechnology, Shanghai, China) and 20% methanol. The number of cells was counted under an inverted microscope (Nikon, Chiyoda-Ku, JP).

For the transwell migration assay, 5 × 10^4^ cells in serum-free medium were seeded into the upper chamber of 8-μm transwell inserts (BD Biosciences, Franklin Lakes, NJ). And medium containing 10% bovine serum albumin was in the lower chamber. After several hours of incubation at 37 °C, cells in the upper chamber were removed carefully and cells adhering to the underside of the membrane were stained with 0.1% crystal violet (Beyotime Institute of Biotechnology, Shanghai, China) and 20% methanol. The number of cells was counted under an inverted microscope (Nikon, Chiyoda-Ku, JP).

All of the above experiments were performed in triplicate. The detailed procedure was as previously described [7, 8].

### Statistical analysis

All data were analyzed using the statistical software SPSS 17 for Windows (SPSS Inc.). The correlation between CTHRC1 expression and clinicopathological parameters was analyzed by Spearman rank-correlation analysis. Survival curves were constructed by Kaplan-Meier method and the difference of these groups were evaluated by using the log-rank test. The Cox proportional hazard regression model was used to identify factors that were independently associated with overall survival and recurrence-free survival. Only factor which was *P* < 0.05 in univariate analysis could be analyzed in multivariate Analysis. Continuous data in this study were presented as mean ± standard deviation (SD) from at least three independent experiments. The differences between groups were analyzed by Student’s *t* test when only two groups were compared or by one-way analysis of variance (ANOVA) when more than two groups were compared. Categorical data were analyzed with χ^2^ test or Fisher’s exact test. A two-tailed *P* < 0.05 was considered statistically significant. Meta-analyses were performed using RevMan 5.3 statistical software. We used Q-test and *I* ^*2*^ test to examine heterogeneity between studies. We used hazard ratio (HR) to evaluate the relationship of CTHRC1 expression with overall survival (OS) and recurrence-free survival (RFS) in breast cancer. To test publication bias, we utilized RevMan 5.3 software to construct a funnel plot. *P* < 0.05 was considered to indicate a significant difference.

## Results

### CTHRC1 is frequently up-regulated in human breast cancer cells and tissues

**Fig. 1:**
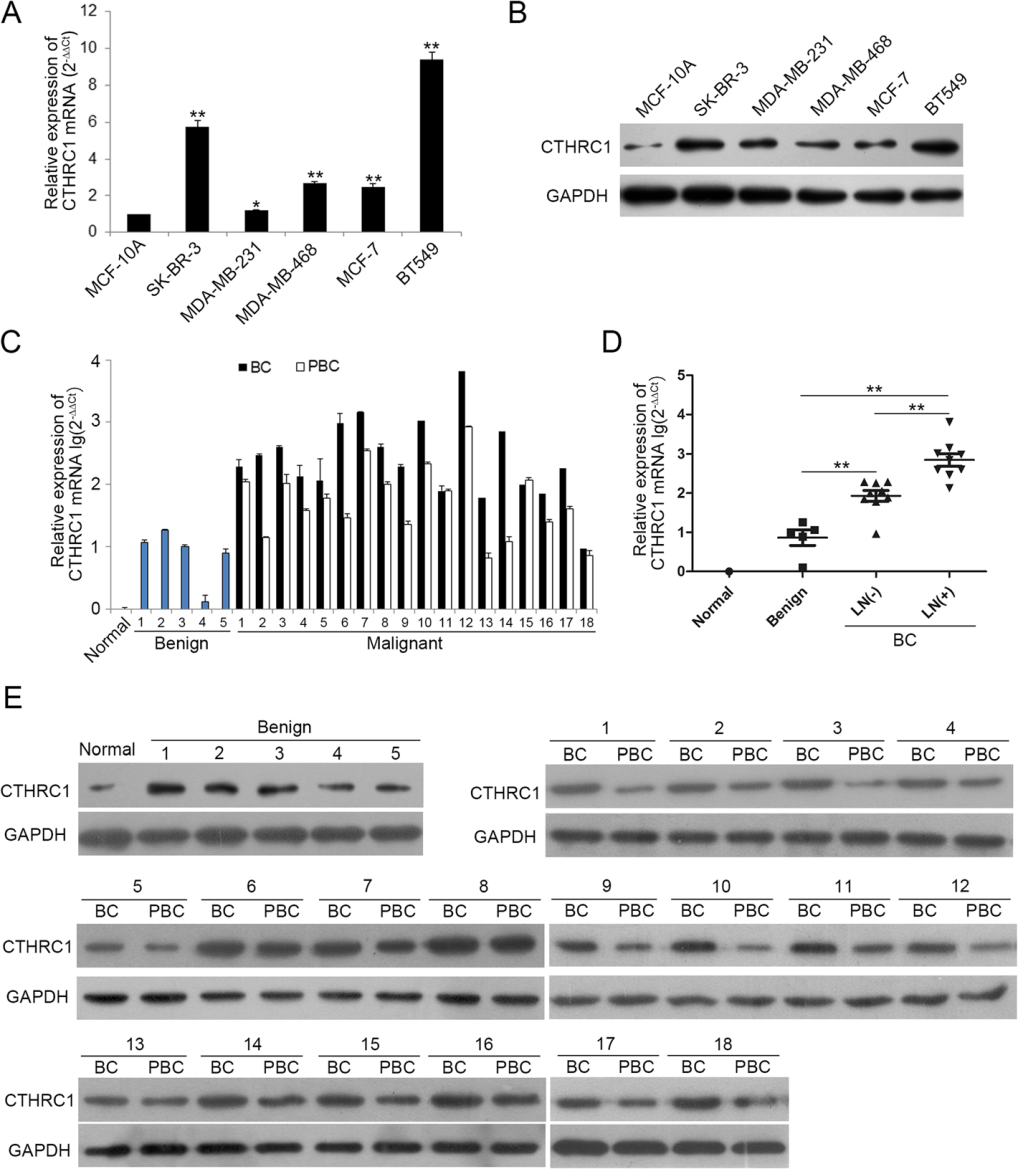
CTHRC1 is frequently up-regulated in human breast cancer cells and tissues. **a** and **b** CTHRC1 was up-regulated in human breast cancer cells analyzed by qRT-PCR and western blot. *P < 0.05, **P < 0.01. **c** CTHRC1 mRNA in normal breast tissue, 5 benign breast tumor tissues and 18 paired breast cancer tissues detected by qRT-PCR. **d** CTHRC1 was significantly step-increased in benign breast tumor tissue, primary breast cancer tissues without LN metastasis and primary breast cancer tissues with LN metastasis. **P < 0.01. **e** CTHRC1 protein in normal breast tissue, 5 benign breast tumor tissues and 18 paired breast cancer tissues detected by western blot

To study the expression of CTHRC1 in human breast cancer cells and tissues, we performed qRT-PCR and western blot. Firstly, CTHRC1 mRNA was detected by qRT-PCR in nontumorigenic MCF-10A breast epithelial cell line and breast cancer cells of different malignance, namely SK-BR-3, MDA-MB-231, MDA-MB-468, MCF-7, and BT549. CTHRC1 mRNA expression level in breast cancer cells was significantly up-regulated, compared to that in MCF-10A (Fig. 1a), which was also verified by western blot (Fig. 1b). Next we detected CTHRC1 mRNA in normal breast tissue, 5 benign breast tumor tissues and 18 paired breast cancer tissues (9 cases with regional lymph node (LN) metastasis and 9 cases without) with qRT-PCR, and results were normalized with its expression in normal tissue. Compared with matched peri-tumor tissue of breast cancer (PBC), CTHRC1 mRNA in breast cancer tissue (BC) was frequently up-regulated (Fig. 1c). Moreover, CTHRC1 mRNA was significantly step-increased in benign breast tumor tissue, primary breast cancer tissues without LN metastasis and primary breast cancer tissues with LN metastasis (Fig. 1d). These results indicated CTHRC1 might promote breast cancer metastasis. We also detected CTHRC1 protein in normal breast tissue, 5 benign breast tumor tissues and 18 paired breast cancer tissues with western blot. Results showed the expression level of CTHRC1 protein was step-increased in normal breast tissue, benign breast tumor tissue and breast cancer tissue, and its expression level in BC was frequently over-expressed in contrast with matched PBC (Fig. 1e). Collectively, all these data indicated CTHRC1 was up-regulated in breast cancer and it may be correlated with breast cancer metastasis.

### High expression of CTHRC1 in breast cancer tissues is associated with aggressive clinicopathological features and lower postoperative survival rate

**Fig. 2:**
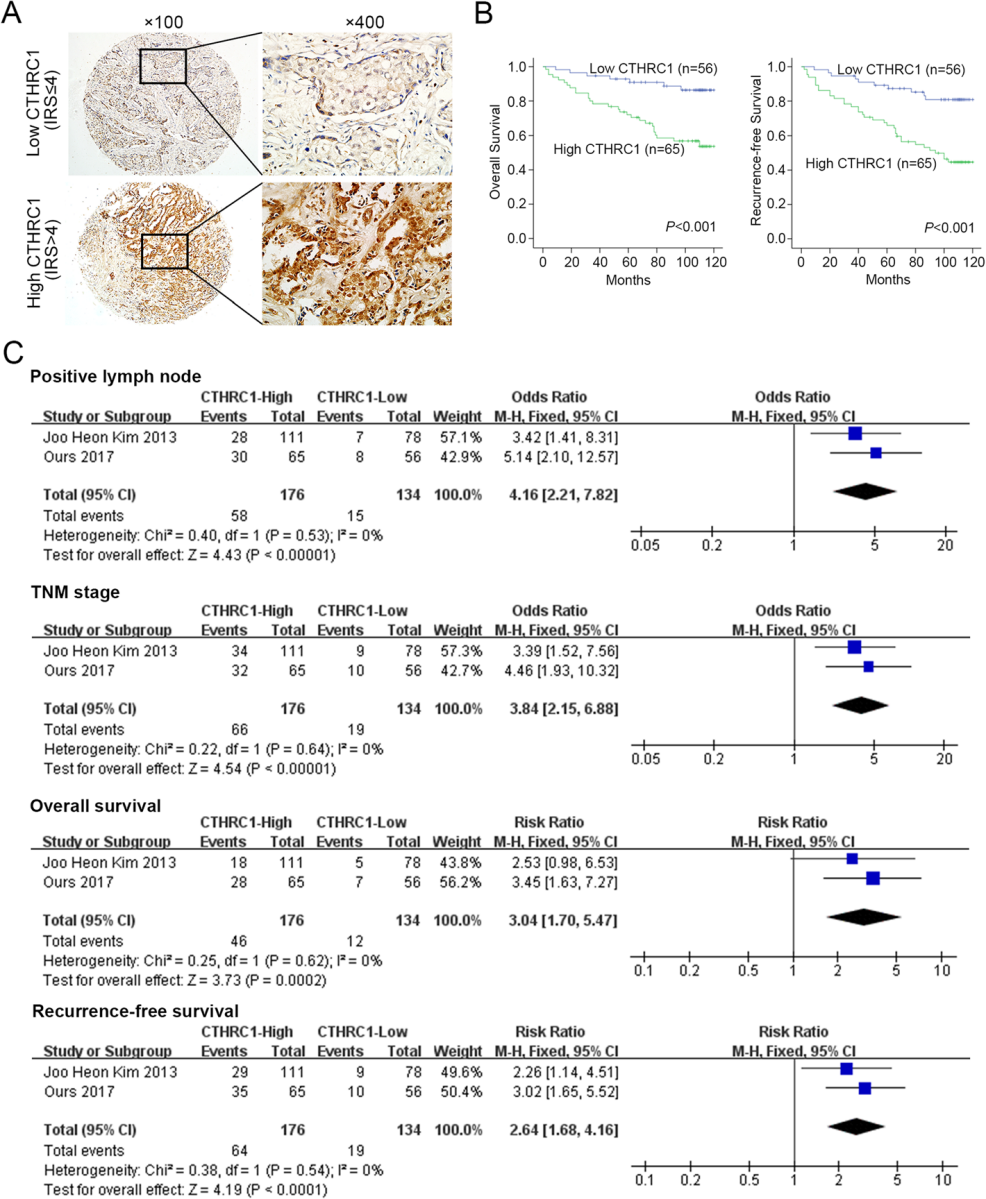
High expression of CTHRC1 in breast cancer tissues is associated with aggressive clinicopathological features and lower postoperative survival rate. **a** Representative immunohistochemistry (IHC) images of CTHRC1 in breast cancer with low/high expression. Magnifications: ×100, ×400. **b** The Kaplan-Meier curves showed overall survival (OS) and recurrence-free survival (RFS) of breast cancer patients with low/high CTHRC1 expression. *P* value was shown in each panel respectively. **c** Forest plots showing the correlation of CTHRC1 with clinical characteristics or OS and RFS

To clarify the clinical relevance of CTHRC1 expression in breast cancer, we performed IHC on breast cancer tissues of 121 breast cancer patients with tissue microarray, and then patients were dichotomized according to low (IRS ≤ 4) or high (IRS > 4) expression of CTHRC1 (Fig. 2a). It showed that the expression level of CTHRC1 was significantly associated with the following clinicopathological features: positive lymph node, lymphovascular invasion, estrogen receptor (ER), progesterone receptor (PR) and TNM stage (all *P* < 0.05, Table 1). We next analyzed the relationship between CTHRC1 expression and the patients’ prognosis. The Kaplan-Meier survival curve demonstrated that patients with high CTHRC1 expression had a shorter overall survival (OS) and recurrence-free survival (RFS) (Both *P* < 0.001, Fig. 2b) than those with low CTHRC1 expression. Moreover, the univariate and multivariate analysis revealed that CTHRC1 expression was an independent prognostic factor for both OS and RFS of breast cancer patients (Tables 2 and 3).

**Table 1:** The Correlations of CTHRC1 with Clinicopathological Features of Breast Cancer Patients

Clinicopathologic Variable	*n*	CTHRC1 expression		*P*
		Low	High	
Age(year)				
≤ 45	35	13	22	
>45	86	43	43	0.198
Histological grade				
I	30	13	17	
II- III	91	43	48	0.709
Positive lymph node				
≤ 3	83	48	35	
>3	38	8	30	*<0.001*
Lymphovascular invasion				
Absence	73	46	27	
Presence	48	10	38	*<0.001*
Tumor size(cm)				
≤ 2	30	16	14	
>2	91	40	51	0.372
ER				
Positive	83	45	38	
Negative	38	11	27	*0.010*
PR				
Positive	57	32	25	
Negative	64	24	40	*0.040*
Her2				
Negative	67	33	34	
Positive	54	23	31	0.465
TNM stage				
I-II	79	46	33	
III	42	10	32	*<0.001*

*Abbreviations*: *ER* estrogen receptor, *PR* progesterone receptor, *Her2* Human Epidermal Growth Factor Receptor type 2, *TNM* tumor node metastasis

*P* ﻿< 0.05 was considered ﻿statistically significant

**Table 2:** Univariate and Multivariate Analysis of Factors Associated with Overall Survival in Breast Cancer Patients

		Univariate Analysis		Multivariate Analysis	
Variable	*n*	RR(95% CI)	*P*	RR(95% CI)	*P*
Age(year)					
≤ 45	35	1			
>45	86	1.416(0.643–3.119)	0.387	n.a.	n.a.
Histological grade					
I	30	1			
II- III	91	2.033(0.789–5.241)	0.142	n.a.	n.a.
Positive lymph node					
≤ 3	83	1		1	
>3	38	2.159(1.110–4.200)	*0.023*	1.546(0.194–12.337)	0.681
Lymphovascular invasion					
Absence	73	1		1	
Presence	48	3.588(1.784–7.216)	*<0.001*	2.507(1.154–5.447)	*0.020*
Tumor size(cm)					
≤ 2	30	1			
>2	91	2.338(0.907–6.031)	0.079	n.a.	n.a.
ER					
Positive	83	1		1	
Negative	38	1.955(1.005–3.804)	*0.048*	1.432(0.706–2.902)	0.320
PR					
Positive	57	1			
Negative	64	1.159(0.593–2.264)	0.666	n.a.	n.a.
Her2					
Negative	67	1		1	
Positive	54	2.279(1.158–4.485)	*0.017*	2.254(1.142–4.449)	*0.019*
TNM stage					
I-II	79	1		1	
III	42	2.123(1.094–4.122)	*0.026*	0.440(0.053–3.667)	0.448
CTHRC1 expression					
Low	56	1		1	
High	65	4.072(1.778–9.329)	*0.001*	2.559(1.017–6.439)	*0.046*

*Abbreviations*: *n.a* Not application, *ER* estrogen receptor, *PR* progesterone receptor, *Her2* Human Epidermal Growth Factor Receptor type 2, *TNM* tumor node metastasis

*P *< 0.05 was considered statistically significant

**Table 3:** Univariate and Multivariate Analysis of Factors Associated with Recurrence-Free Survival in Breast Cancer Patients

		Univariate Analysis		Multivariate Analysis	
Variable	*n*	RR(95% CI)	*P*	RR(95% CI)	*P*
Age(year)					
≤ 45	35	1			
>45	86	1.033(0.542–1.968)	0.922	n.a.	n.a.
Histological grade					
I	30	1			
II- III	91	1.905(0.851–4.266)	0.117	n.a.	n.a.
Positive lymph node					
≤ 3	83	1		1	
>3	38	2.282(1.270–4.102)	*0.006*	1.699(0.223–12.925)	0.609
Lymphovascular invasion					
Absence	73	1		1	
Presence	48	3.192(1.746–5.837)	*<0.001*	2.245(1.150–4.385)	*0.018*
Tumor size(cm)					
≤ 2	30	1			
>2	91	1.385(0.686–2.799)	0.364	n.a.	n.a.
ER					
Positive	83	1			
Negative	38	1.568(0.863–2.848)	0.140	n.a.	n.a.
PR					
Positive	57	1			
Negative	64	1.099(0.610–1.978)	0.754	n.a.	n.a.
Her2					
Negative	67	1		1	
Positive	54	1.942(1.078–3.499)	*0.027*	1.944(1.077–3.507)	*0.027*
TNM stage					
I-II	79	1		1	
III	42	2.192(1.221–3.936)	*0.009*	0.477(0.059–3.831)	0.486
CTHRC1 expression					
Low	56	1		1	
High	65	3.674(1.818–7.425)	*<0.001*	2.490(1.140–5.441)	*0.022*

*Abbreviations*: *n.a* Not application, *ER* estrogen receptor, *PR* progesterone receptor, *Her2* Human Epidermal Growth Factor Receptor type 2, *TNM* tumor node metastasis

*P *< 0.05 was considered statistically significant

As Kim et al. [11] have investigated the clinical significance of CTHRC1 expression in breast cancers from 189 patients by immunohistochemistry and reported CTHRC1 over-expression might indicate a poor clinical outcome, combined with their results, we performed a meta-analysis to further corroborate the prognostic value of CTHRC1. The basic characteristics of their article and ours were listed in Additional file 1: Table S1. We adopted the following parameters: positive lymph node, TNM stage, ER, PR, OS and RFS. A total of 310 breast cancer patients were included in this meta-analysis, of which 176 were of high CTHRC1 expression and 134 were of low expression. We found high CTHRC1 expression was significantly associated with the following clinicopathological characteristics: positive lymph node (OR = 4.16, 95% CI = [2.21, 7.82], *P* < 0.00001, fixed-effect), and TNM stage (OR = 3.84, 95% CI = [2.15, 6.88], *P* < 0.00001, fixed-effect) (Fig. 2c). Moreover, high CTHRC1 expression was not markedly associated with ER (OR = 1.31, 95% CI = [0.29, 5.91], *P* = 0.73, random-effect) and PR (OR = 1.10, 95% CI = [0.31, 3.88], *P* = 0.88, random-effect) (Additional file 3: Figure S1A). We also found high CTHRC1 expression was significantly associated with poor OS (RR = 3.04, 95% CI = [1.70, 5.47], *P* = 0.0002, fixed-effect) and RFS (RR = 2.64, 95% CI = [1.68, 4.16], *P* < 0.0001, fixed-effect) in breast cancer patients (Fig. 2c). Funnel plots indicated publication bias was excluded and the results were credible (Additional file 3: Figure S1B).

Taken together, these data fully demonstrated that CTHRC1 was closely correlated with poor survival and might be used as a novel independent prognosis biomarker for breast cancer.

### CTHRC1 and miR-30c expression are inversely correlated in human breast cancer cells and tissues

**Fig. 3:**
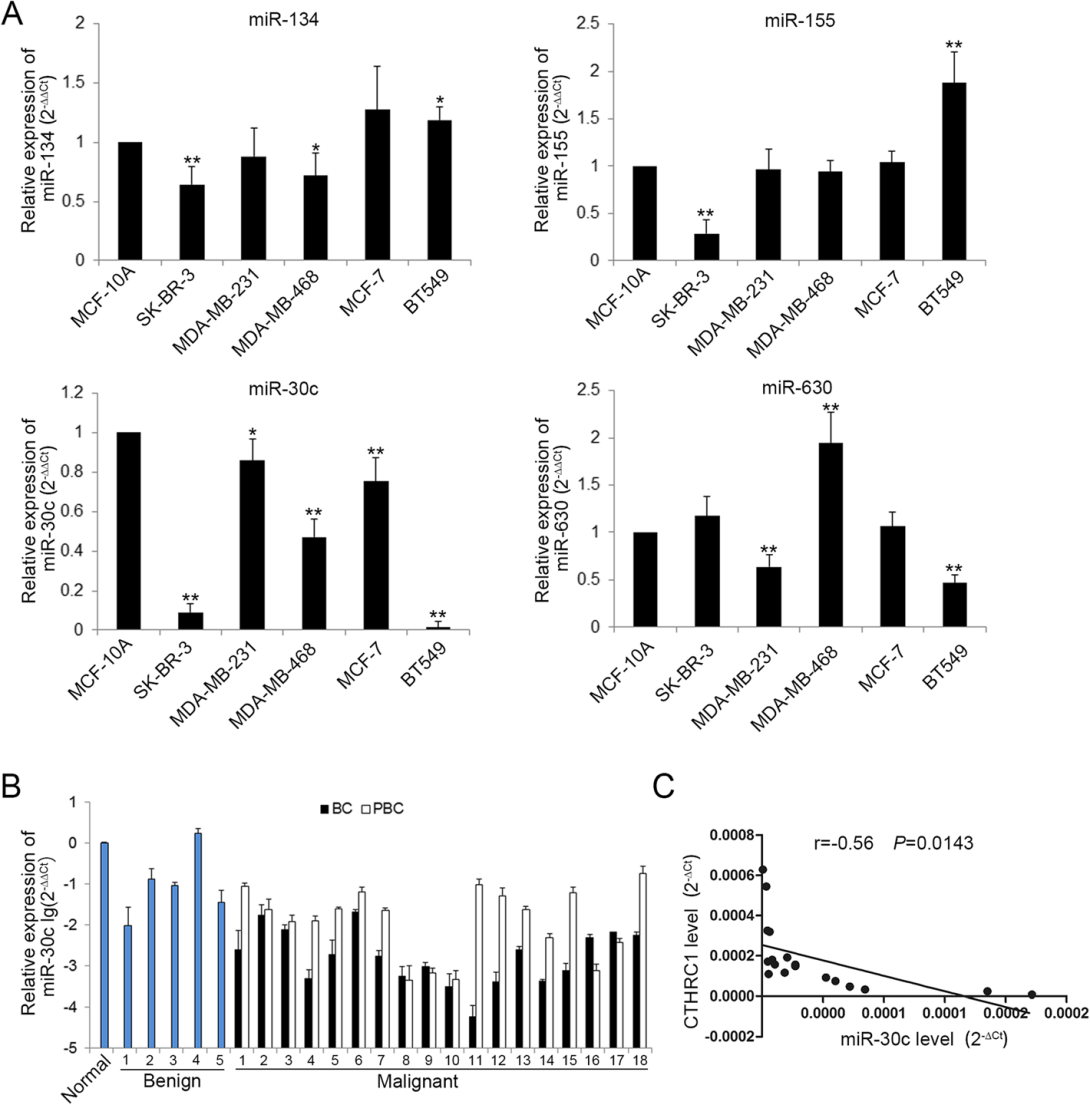
CTHRC1 and miR-30c expression are inversely correlated in human breast cancer cells and tissues. **a** The relative expression level of miR-134, miR-155, miR-30c and miR-630 in breast cancer cells respectively was detected by qRT-PCR. *P < 0.05, **P < 0.01. **b** The relative expression level of miR-30c in normal breast tissue, 5 benign breast tumor tissues and 18 paired breast cancer tissues was detected by qRT-PCR. **c** Correlation analysis of miR-30c expression and CTHRC1 expression in clinical breast cancer samples. *r* = −0.56, *P* = 0.0143

Next, we investigated the mechanism that regulates CTHRC1 expression. Since microRNAs are master regulators of gene expression and play important role in tumor progression [17], we sought to identify miRNAs that regulate CTHRC1 expression. We used miRwalk database to identify potential miRNAs that bind to 3′ UTR of CTHRC1 and identified miR-134, miR-155, miR-30c and miR-630 as possible candidates (Additional file 4: Figure S2A). Then we investigated the expression of these candidate miRNAs in nontumorigenic breast epithelial cell line MCF-10A and breast cancer cells by qRT-PCR and found only miR-30c was markedly down-regulated in breast cancer cells compared to MCF-10A (Fig. 3a). Moreover, our previous miRNA microarray analysis also revealed that miR-30c was significantly down-regulated in breast cancer tissues (Additional file 4: Figure S2B). In addition, Rodr et al. [18] and Bockhorn et al. [19] have successively reported the low expression of miR-30c was associated to poor prognosis in breast cancer, whereas our data showed CTHRC1 high-expression indicated poor prognosis. All of these further prompted us to postulate miR-30c was potential critical upstream negative regulator of CTHRC1. Therefore, we focused on miR-30c for further study. We detected miR-30c in normal breast tissue, 5 benign breast tumor tissues and 18 paired breast cancer tissues with qRT-PCR, and results were normalized with its expression in normal tissue. Compared with matched PBC, miR-30c in BC was frequently down-regulated (Fig. 3b). Furthermore, there was an inverse correlation between the expression of miR-30c and CTHRC1 in breast cancer tissues (Fig. 3c, r = −0.56, *P* = 0.0143). Thus, these data indicated that loss of miR-30c was related to the up-regulation of CTHRC1.

### CTHRC1 is a direct target of miR-30c

**Fig. 4:**
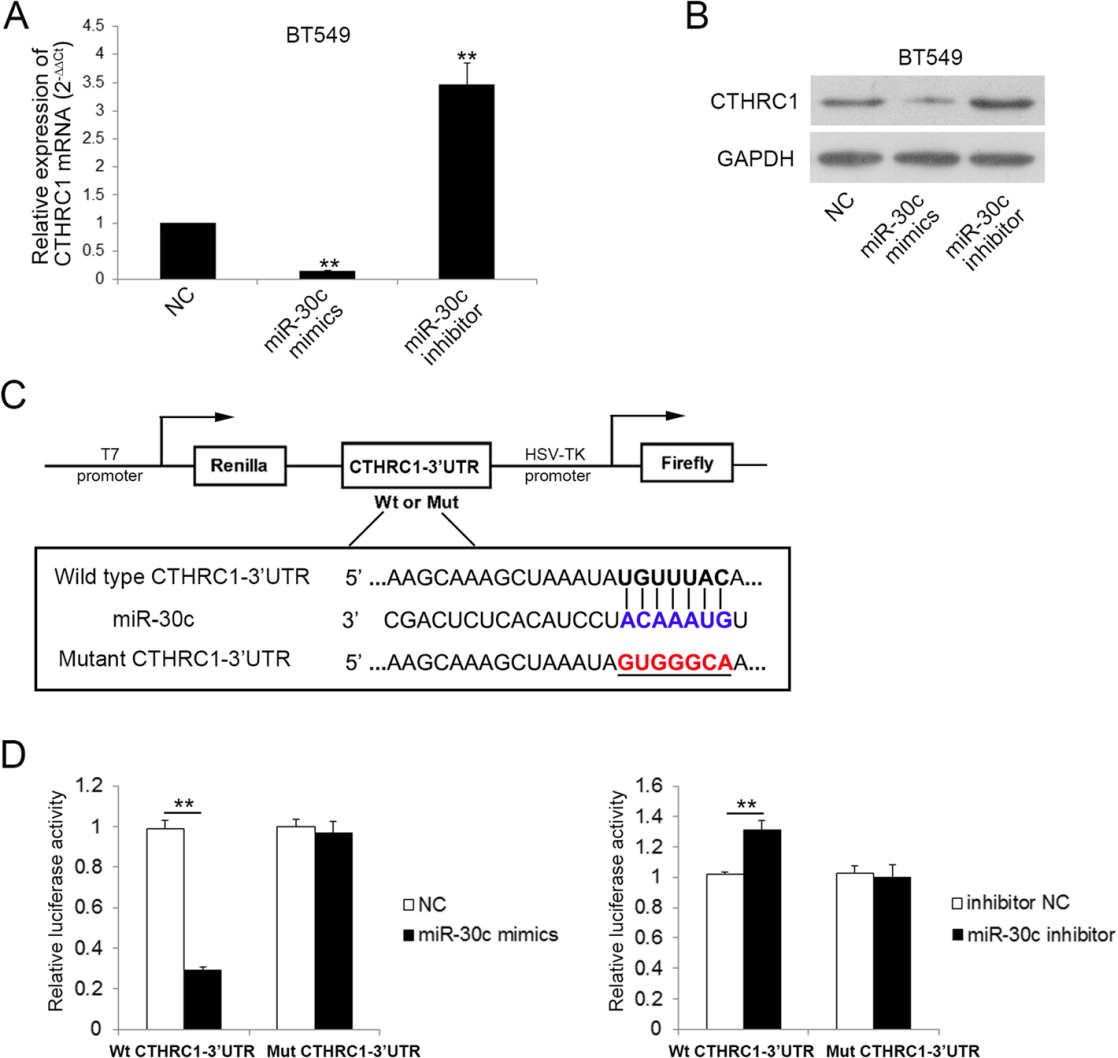
CTHRC1 is a direct target of miR-30c. **a** qRT-PCR analysis of CTHRC1 mRNA expression in indicated cells 24 h post-transfection. **P < 0.01. **b** CTHRC1 protein expression was detected by western blot in indicated cells post-transfection. **c** Wild type (Wt) and Mutant type (Mut) CTHRC1 3’UTR sequences were cloned into a psi-CHECK2 reporter vector. **d** The relative luciferase activity was detected by dual-luciferase reporter assay in indicated cells. **P < 0.01

To determine whether CTHRC1 is a direct downstream target of miR-30c, we firstly transfected miR-30c mimics or miR-30c inhibitor into BT549 cells, and then detected CTHRC1 expression level with qRT-PCR and western blot. Results showed gain of miR-30c decreased both mRNA and protein level of CTHRC1, and loss of miR-30c caused up-regulation of CTHRC1 (Fig. 4a, b). Next we cloned wild-type and mutant CTHRC1–3′ UTR target sequences into the luciferase reporter vector (Fig. 4c) and transfected into HEK293T cells with miR-30c mimics or inhibitor also transfected. We found miR-30c mimics markedly decreased the luciferase activity of Wt 3′ UTR of CTHRC1, whereas miR-30c inhibitor up-regulated the luciferase activity; and the luciferase activity of Mut 3′ UTR of CTHRC1 showed no significant difference (Fig. 4d). Taken together, these results demonstrated that CTHRC1 was directly regulated by miR-30c.

### Ectopic expression of miR-30c or gain and loss of CTHRC1 affects breast cancer cell proliferation, apoptosis, invasion and migration

**Fig. 5:**
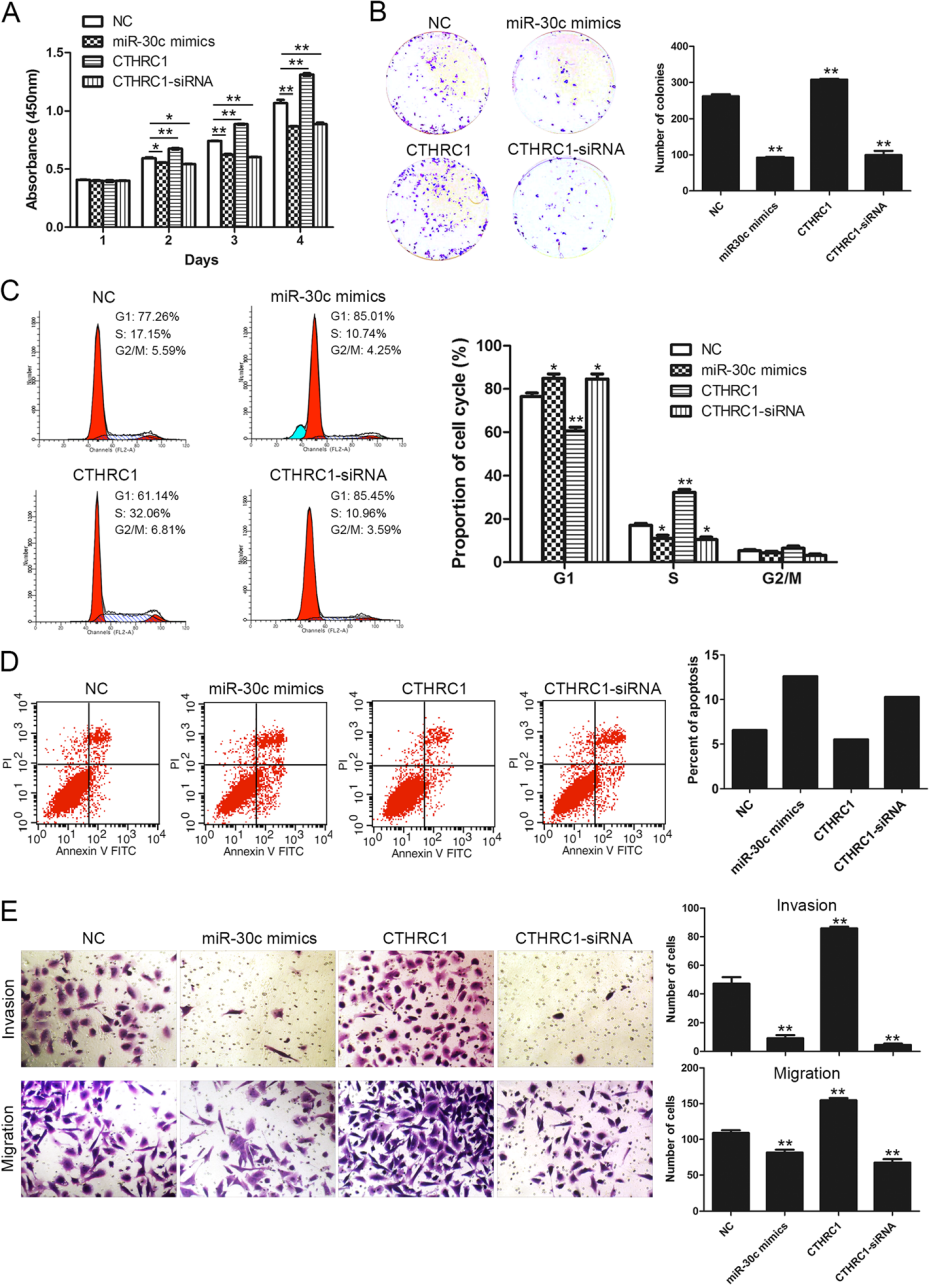
Ectopic expression of miR-30c or gain and loss of CTHRC1 affects breast cancer cell proliferation, apoptosis, invasion and migration. **a** The effect of ectopic expression of miR-30c or gain and loss of CTHRC1 on cell viability was detected by CCK-8 assay. *P < 0.05, **P < 0.01. **b** The effect of ectopic expression of miR-30c or gain and loss of CTHRC1 on cell proliferation was measured by colony formation assay. Representative images of colony formation are shown (left panel). Results represented the mean ± SD in triplicate using bar graph (right panel). **P < 0.01. **c** The effect of ectopic expression of miR-30c or gain and loss of CTHRC1 on cell cycle progression was measured by flow cytometry analysis. *P < 0.05, **P < 0.01. **d** The effect of ectopic expression of miR-30c or gain and loss of CTHRC1 on cell apoptosis was detected by flow cytometry analysis. **e** The effect of ectopic expression of miR-30c or gain and loss of CTHRC1 on cell invasion and migration was measured by transwell invasion/migration assay. Representative images are shown (left panel). Magnification: ×100. Results represented the mean ± SD in triplicate using bar graph (right panel). **P < 0.01

The above results promoted us to further explore the biological functions of miR-30c/CTHRC1 axis in BT549 cells. We firstly performed CCK8 assay to investigate its role in cell proliferation. Results demonstrated ectopic expression of miR-30c resulted in a markedly decreased cell viability, which could be mimicked by loss of CTHRC1 with CTHRC1-siRNA, whereas gain of CTHRC1 significantly increased cell viability (Fig. 5a). We further adopted colony formation assay and found restoration of miR-30c markedly decreased the number of colonies, which could be mimicked by knock-down of CTHRC1, whereas overexpression of CTHRC1 significantly increased the number of colonies (Fig. 5b). Also, cell cycle analysis revealed a significant increase in the percentage of cells in G1 phase and a decrease in the percentage of cells in S phase in cells transfected with miR-30c, which could be mimicked by CTHRC1 knock-down, whereas gain of CTHRC1 decreased the proportion of cells in G1 phase and increased the proportion of cells in S phase (Fig. 5c). Next we explored the role of miR-30c/CTHRC1 axis in cell apoptosis. Flow cytometry revealed that ectopic expression of miR-30c markedly increased cell apoptosis rate, which could be mimicked by loss of CTHRC1, whereas gain of CTHRC1 decreased apoptosis rate (Fig. 5d). Finally, we studied its function on cell invasion and migration. Transwell invasion/migration assay demonstrated restoration of miR-30c markedly suppressed invasion and migration of BT549 cells, which could be mimicked by knock-down of CTHRC1, whereas overexpression of CTHRC1 significantly increased cell invasion and migration (Fig. 5e). Therefore, these data indicated CTHRC1, which was negatively regulated by miR-30c, promoted cell proliferation, invasion and migration, and inhibited cell apoptosis.

### Ectopic expression of miR-30c or gain and loss of CTHRC1 affects GSK-3β/β-catenin signaling and Bax/Caspase-9/Caspase-3 signaling in breast cancer

**Fig. 6:**
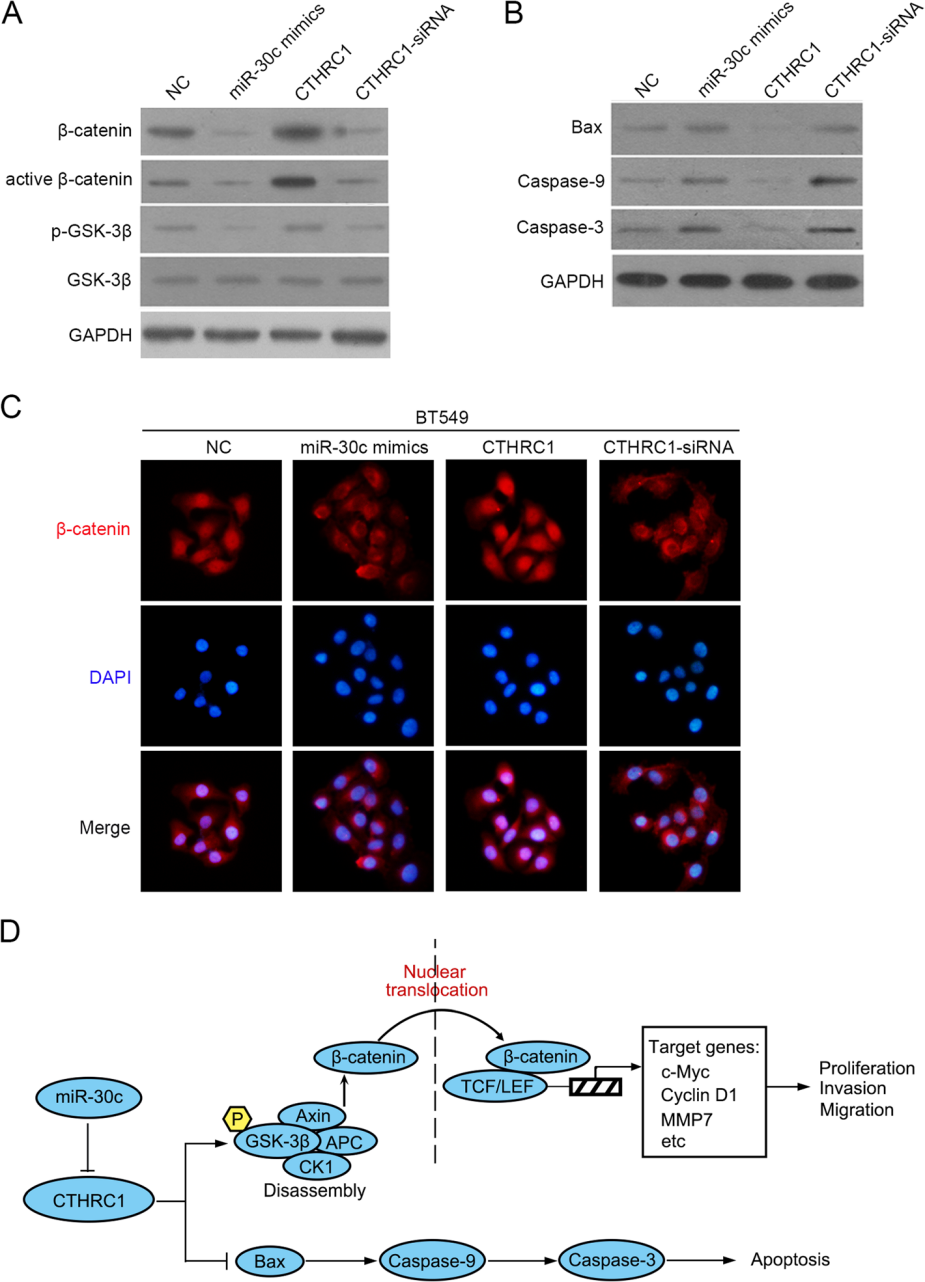
Ectopic expression of miR-30c or gain and loss of CTHRC1 affects GSK-3β/β-catenin signaling and Bax/Caspase-9/Caspase-3 signaling in breast cancer. **a** Protein levels of β-catenin, active β-catenin, normal and phosphorylated form of GSK-3β detected by western blot were shown in indicated cells. **b** Protein levels of Bax, Caspase-9 and Caspase-3 detected by western blot were shown in indicated cells. **c** Immunofluorescence staining of β-catenin in indicated cells. Original magnification: ×100. **d** Schematic representation of the major molecular mechanism that miR-30c/CTHRC1 axis exerts its role in cell proliferation, apoptosis, invasion and migration

We previously have found that CTHRC1 promotes NSCLC cell aggressiveness by targeting GSK-3β/β-catenin pathway [7]. Therefore, we hypothesized miR-30c/CTHRC1 axis might also exert its role by targeting GSK-3β/β-catenin signaling in breast cancer. We firstly detected β-catenin and its active (dephosphorylated) form with western blot, and found ectopic expression of miR-30c resulted in a markedly decrease of β-catenin and its active form, which could be mimicked by loss of CTHRC1 with CTHRC1-siRNA, whereas gain of CTHRC1 significantly increased β-catenin and its active form (Fig. 6a). To find out in which way β-catenin were activated, we continued to measure normal and phosphorylated form of glycogen synthase kinase 3β (GSK-3β), which was upstream regulator of β-catenin in Wnt/β-catenin signaling. Results showed restoration of miR-30c markedly suppressed the phosphorylation of GSK-3β at Ser9, which could be mimicked by knock-down of CTHRC1, whereas overexpression of CTHRC1 significantly promoted the phosphorylation of GSK-3β (Fig. 6a). Meanwhile, we explored the mechanism of its role in apoptosis. We focused on Bax, a vital cell death regulator, is an indispensable gateway to mitochondrial dysfunction and a major proapoptotic member of the B-cell lymphoma 2 (Bcl-2) family proteins that control apoptosis in normal and cancer cells [20–22]. We thus detected Bax with western blot and found ectopic expression of miR-30c markedly increased the expression level of Bax, which could be mimicked by loss of CTHRC1, whereas gain of CTHRC1 decreased Bax expression (Fig. 6b). Since both caspase-9 and caspase-3 are putative downstream factors of Bax [23], we next examined their expression levels. Western analyses showed that the caspase-9 and caspase-3 were significantly elevated after restoration of miR-30c, which was mimicked in the group with CTHRC1 knock-down, whereas gain of CTHRC1 markedly down-regulated caspase-9 and caspase-3 (Fig. 6b). Furthermore, as β-catenin’s nuclear translocation was the hallmark of activating Wnt/β-catenin signaling [24], we performed immunofluorescence for cells to further corroborate the nuclear localization of β-catenin. Results demonstrated the β-catenin in nucleus was decreased evidently after ectopic expression of miR-30c, which was mimicked by loss of CTHRC1, whereas gain of CTHRC1 enhanced the nuclear localization of β-catenin (Fig. 6c).

Taken together, the above data indicated CTHRC1, negatively regulated by miR-30c, might promote cell proliferation, invasion and migration by activating GSK-3β/β-catenin signaling and suppress cell apoptosis by inhibiting Bax/Caspase-9/Caspase-3 signaling (Fig. 6d).

## Discussion

In breast cancer, around 15% of patients develop disseminated metastasis before or after diagnosis, and distant metastasis is responsible for approximately 90% of breast cancer-associated mortality [25]. Activating invasion and metastasis is an important hallmark of cancer [26]. The multistep process of invasion and metastasis has been schematized as a sequence of discrete steps, often termed the invasion-metastasis cascade [27]. Research into the capability for invasion and metastasis has accelerated dramatically over the past decade and resulted in the development of hallmark-targeting therapies [28]. However, a targeted therapeutic agent inhibiting one key pathway in a tumor may not completely shut off a hallmark capability, allowing some cancer cells to survive with residual function until they or their progeny eventually adapt to the selective pressure imposed by the therapy being applied [26, 28]. Therefore, identifying more key regulators in cancer metastasis and targeting all of these supporting pathways therapeutically become increasingly important. In this study, we found CTHRC1 promoted cell proliferation, invasion and migration and suppressed cell apoptosis in breast cancer, which might be by activating GSK-3β/β-catenin signaling and inhibiting Bax/Caspase-9/Caspase-3 signaling respectively; and these biological functions of CTHRC1 could be directly negatively regulated by miR-30c. In addition, our cohort study and further meta-analysis validated high expression of CTHRC1 was associated with aggressive clinicopathological features and poor clinical outcome of breast cancer patients. Yet there still exist some potential problems to be further discussed and explored.

Wnt/β-catenin signaling plays a critical role in a variety of biological processes, including cell proliferation, migration, polarity establishment and stem cell self-renewal [24, 29]. Deregulation of Wnt/β-catenin signaling is associated with development of various cancers, including breast cancer [30, 31]. Wnt ligands initiate this signaling by interacting with cell surface receptors Frizzled (Fz) and low-density lipoprotein receptor-related protein 6 (LRP6). Subsequent recruitment of the downstream signal mediators Dvl to Fz and Axin to LRP6 results in disassembly of the β-catenin destruction complex consisting of Axin, adenomatous polyposis coli (APC), GSK-3β and casein kinase 1 (CK1), then leading to the nuclear translocation of β-catenin. Binding of β-catenin with the T-cell factor/lymphoid enhancer-binding factor (TCF/LEF) activates the transcription of Wnt target genes, such as c-Myc, Cyclin D1 and MMP7, ultimately initiating cell proliferation, invasion and migration [32, 33]. In this study we presented evidence that CTHRC1 could promote GSK-3β phosphorylation at Ser9 and thus GSK3β-mediated phosphorylation of β-catenin at Ser-33, Ser-37, and Thr-41 [34] was inhibited. Therefore, ubiquitin-dependent degradation of β-catenin was prevented, after which β-catenin accumulated in cytoplasm and then translocated into nuclear, thereby activating this signaling for cell proliferation and metastasis. However, how CTHRC1 promoted phosphorylation of GSK-3β remains to be further studied. Furthermore, it has been widely recognized that the activation of Wnt/β-catenin signaling can induce epithelial-mesenchymal transition (EMT). EMT is a vital initiator of and a contributor to tumor metastasis, during which, polarized epithelial cells lost their cell-cell junctions and converted into individual, non-polarized, motile and invasive mesenchymal cells [35]. Accumulating researches have focused on the role of EMT in breast cancer progression, but still remain inconclusive [36–38]. In this study, we also found it seemed that interfering the expression of CTHRC1 might change the morphology of BT549 cells when performing cell immunofluorescence, indicating CTHRC1 might also regulate EMT of breast cancer cells. Thus in future study, we will also focus on whether CTHRC1 regulates breast cancer progression by targeting EMT.

In addition, we found CTHRC1 could suppress breast cancer cell apoptosis. Apoptosis is a form of programmed cell death that occurs in a physiological setting and is indispensable for the preservation of tissue homeostasis and embryonic development [26, 39]. Importantly, apoptosis down-regulation is a major cause of tumorigenesis, cancer progression and chemo-resistance in various cancers, including breast cancer [40, 41]. The apoptotic machinery is composed of upstream regulators and downstream effectors, and controlled by counterbalancing pro- and antiapoptotic members of the Bcl-2 family of regulatory proteins [42]. The archetype, Bcl-2, along with its closest relatives (Bcl-xL, etc.) are inhibitors of apoptosis, acting in large part by binding to and thereby suppressing two proapoptotic triggering proteins (Bax and Bak) [22]. When relieved of inhibition by antiapoptotic relatives, Bax and Bak disrupt the integrity of the outer mitochondrial membrane, causing the release of proapoptotic signaling proteins, key of which is cytochrome C. Then a cascade of caspases (Caspase-9, Caspase-3) is activated, that act via their proteolytic activities to induce the multiple cellular changes associated with the apoptotic program [23, 43]. In this study, we identified CTHRC1 could down-regulate Bax and thus inhibit the activation of Caspase-9 and Caspase-3. However, exactly how CTHRC1 inactivates the Bax/Caspase-9/Caspase-3 pathway needs to be further explored.

Last but not least, in this study we identified CTHRC1 as an independent prognostic factor for breast cancer patients, suggesting it might be used for molecular classification of breast cancer. Precision medicine is about matching the right drugs to the right patients [44]. We supposed those breast cancer patients with CTHRC1 high-expression could be characterized into poor prognosis group, whose therapeutic effect of traditional strategies was not so good, and might be treated with strategies designed to up-regulate miR-30c. The potential of miRNAs in regulating different cellular pathways and as mediators of interactions between cells make them ideal drug targets. MicroRNA-based strategies for anti-cancer therapy are becoming increasingly possible, and promising results have been achieved with the use of miRNA antagonists and miRNA mimics in preclinical studies. Moreover, due to advances in the understanding of specific miRNA modulatory role in immune system and tumor-mediated immunity, researchers are also developing novel miRNA-based interventions for cancer immunotherapy [45]. However, successful delivery directed to specific tissue and cell, represents a big challenge for in vivo miRNA-mediated anti-cancer therapy.

## Conclusion

Our data demonstrated that CTHRC1, negatively regulated by miR-30c, promoted cell proliferation, invasion and migration and suppressed cell apoptosis in breast cancer, which might be by activating GSK-3β/β-catenin signaling and inhibiting Bax/Caspase-9/Caspase-3 signaling respectively. Furthermore, our cohort study and further meta-analysis validated CTHRC1 was an independent prognostic indicator for breast cancer patients. Therefore, we suppose strategies designed to up-regulate miR-30c or down-regulate CTHRC1 may provide a promising method to alleviate breast cancer progression.

## Additional files

Additional file 1: Table S1. The basic characteristics of the included studies. **Table S2** The sequences of qRT-PCR primers used in this study. **Table S3** List of the antibodies used in this study. **Table S4** List of the reagents used in this study. **Table S5** List of the sequences used in this study. (DOC 68 kb)

Additional file 2: Figure S3. To confirm the interference efficiency on miR-30c and CTHRC1. A, The relative expression level of miR-30c overexpression and inhibition in indicated cells was detected by qRT-PCR. **P < 0.01. B, The relative expression level of CTHRC1 overexpression in indicated cells was detected by qRT-PCR. **P < 0.01. C, Silencing efficiency of CTHRC1 in mRNA level by siRNA-1, −2 and −3 in indicated cells were identified by qRT-PCR. **P < 0.01. D, Overexpression and silencing efficiency of CTHRC1 in protein level were identified by western blot. (TIFF 503 kb)

Additional file 3: Figure S1. Meta-analysis on prognostic value of CTHRC1. A, Forest plots showing the correlation of CTHRC1 with ER and PR. B, Funnel plots for publication bias. (TIFF 527 kb)

Additional file 4: Figure S2. To identify miRNAs that regulate CTHRC1 expression. A, miRwalk database identified potential miRNAs that bind to 3′ UTR of CTHRC1. B, miRNA microarray analysis revealed miR-30c was significantly down-regulated in breast cancer tissues. (TIFF 689 kb)

## Abbreviations

APC: Adenomatous polyposis coli
BC: Breast cancer
CK1: Casein kinase 1
CTHRC1: Collagen triple helix repeat containing-1
DCIS: Ductal carcinoma in situ
Dvl: Dishevelled
ER: Estrogen receptor
GSK-3β: Glycogen synthase kinase 3β
Her2: Human Epidermal Growth Factor Receptor type 2
IHC: Immunohistochemistry
IRS: Immunoreactive score
LRP6: Low-density lipoprotein receptor-related protein 6
OS: Overall survival
PBC: Peri-tumor tissue of breast cancer
PR: Progesterone receptor
qRT-PCR: Quantitative real-time PCR
RFS: Recurrence-free survival
RT-PCR: Reverse-transcription polymerase chain reaction
TCF/LEF: T-cell factor/lymphoid enhancer-binding factor
TNM: Tumor node metastasis
