# Supplementary material for: Collagen triple helix repeat containing-1 negatively regulated by microRNA-30c promotes cell proliferation and metastasis and indicates poor prognosis in breast cancer
Source: J Exp Clin Cancer Res. 2017 Jul 12;36:92. doi: 10.1186/s13046-017-0564-7 (PMC5506643; doi:10.1186/s13046-017-0564-7)
Supplement: Supplementary file 1 — The basic characteristics of the included studies. Table S2 The sequences of qRT-PCR primers used in this study. Table S3 List of the antibodies used in this study. Table S4 List of the reagents used in this study. Table S5 List of the sequences used in this study. (DOC 68 kb) [file 13046_2017_564_MOESM1_ESM.doc]

**Supplementary Tables**

**Supplementary Table 1. The basic characteristics of the included studies.**

| Author | Year | Country | Method | Total | CTHRC1-High(n) | CTHRC1-Low(n) |
| --- | --- | --- | --- | --- | --- | --- |
| Joo Heon Kim | 2013 | Korea | IHC | 189 | 111 | 78 |
| Ours | 2017 | China | IHC | 121 | 65 | 56 |

**Supplementary Table 2. The sequences of qRT-**PCR primers used in this study

| Target gene | Sequence |
| --- | --- |
| CTHRC1 | F: ATAATGGAATGTGCTTACAAGG |
|  | R: TTCCCAAGATCTATGCCATAAT |
| 18SrRNA | F: CCTGGATACCGCAGCTAGGA |
|  | R: GCGGCGCAATACGAATGCCCC |
| miR-134 | RT:CTCAACTGGTGTCGTGGAGTCGGCAATTCAGTTGAGCCCCTCTG |
|  | F: ACACTCCAGCTGTGACTGGTTGACCAGA |
|  | R: CTCAACTGGTGTCGTGGA |
| miR-155 | RT:CTCAACTGGTGTCGTGGAGTCGGCAATTCAGTTGAGACCCCTA |
|  | F: ACACTCCAGCTTAATGCTAATCGTGATAG |
|  | R: CTCAACTGGTGTCGTGGA |
| miR-30c | RT:CTCAACTGGTGTCGTGGAGTCGGCAATTCAGTTGAGGCTGAGA |
|  | F: ACACTCCAGCTGTAAACATCCTACACTCT |
|  | R: CTCAACTGGTGTCGTGGA |
| miR-630 | RT:CTCAACTGGTGTCGTGGAGTCGGCAATTCAGTTGAGACCTTCC |
|  | F: ACACTCCAGCAGTATTCTGTACCAGG |
|  | R: CTCAACTGGTGTCGTGGA |
| U6 | RT:CTCAACTGGTGTCGTGGAGTCGGCAATTCAGTTGAGAAAAATATGG |
|  | F: CTCGCTTCGGCAGCACA |
|  | R: AACGCTTCACGAATTTGCGT |

**Supplementary Table 3**. List of the antibodies used in this study.

| Antibody name | Source |
| --- | --- |
| CTHRC1 | Abcam (ab85739) |
| GAPDH | KangChen (KC-5G5) |
| β-catenin | Santa Cruz (sc-133239) |
| Active β-catenin | Cell signaling (8814) |
| GSK-3β | Abcam (ab32391) |
| p- GSK-3β | Abcam (ab75745) |
| Bax | Abcam (ab32503) |
| Caspase-9 | Abcam (ab202068) |
| Caspase-3 | Abcam (ab13847) |

**Supplementary Table 4**. List of the reagents used in this study.

| Reagent name | Source |
| --- | --- |
| Donkey anti-Mouse IgG (H+L) Cross Adsorbed Secondary Antibody, DyLight 594 conjugate | ThermoFisher Scientific (SA5-10168) |
| Anti-Mouse IgG (whole molecule)–Peroxidase antibody produced in goat | Sigma (A5278) |
| Goat anti-Rabbit IgG-HRP Secondary Antibody | ZSGB-BIO (ZDR-5306) |
| Polymer HRP Detection System | ZSGB-BIO (PV-9000) |
| DAB Kit | ZSGB-BIO (ZLI-9018) |
| Dual-Luciferase Reporter Assay Kit | Promega (E1910) |

**Supplementary Table 5. List of the sequences used in this study.**

| Name | Sequence |
| --- | --- |
| miR-30c mimics | UGUAAACAUCCUACACUCUCAGC |
| NC | UUUGUACUACACAAAAGUACUG |
| miR-30c inhibitor | UGUAAACAUCCUACACUCUCAGC |
| Inhibitor NC | UUUGUACUACACAAAAGUACUG |
| CTHRC1-siRNA-1 | CGGAGUGUACAUUUACAAATT |
| CTHRC1-siRNA-2 | CCAUUGAAGCUAUCAUCUATT |
| CTHRC1-siRNA-3 | CGCAUCAUUAUUGAAGAACUATT |
| ORF nucleotide sequence of CTHRC1 | ATGTGGCCGCCAGGTAGGAGCATCACAGTCAAGCTACGGGAGAAAACAGTTTCCAGGAAACTGGAAATGAACGGCCCGAGTGCTTTCCAGGGGCTCATCTGTGGGAAGTATAATGGAATGTGCTTACAAGGGCCAGCAGGAGTGCCTGGTCGAGACGGGAGCCCTGGGGCCAATGGCATTCCGGGTACACCTGGGATCCCAGGTCGGGATGGATTCAAAGGAGAAAAGGGGGAATGTCTGAGGGAAAGCTTTGAGGAGTCCTGGACACCCAACTACAAGCAGTGTTCATGGAGTTCATTGAATTATGGCATAGATCTTGGGAAAATTGCGGAGTGTACATTTACAAAGATGCGTTCAAATAGTGCTCTAAGAGTTTTGTTCAGTGGCTCACTTCGGCTAAAATGCAGAAATGCATGCTGTCAGCGTTGGTATTTCACATTCAATGGAGCTGAATGTTCAGGACCTCTTCCCATTGAAGCTATAATTTATTTGGACCAAGGAAGCCCTGAAATGAATTCAACAATTAATATTCATCGCACTTCTTCTGTGGAAGGACTTTGTGAAGGAATTGGTGCTGGATTAGTGGATGTTGCTATCTGGGTTGGTACTTGTTCAGATTACCCAAAAGGAGATGCTTCTACTGGATGGAATTCAGTTTCTCGCATCATTATTGAAGAACTACCAAAATAA |
