# Supplementary figures and images for: Collagen triple helix repeat containing-1 negatively regulated by microRNA-30c promotes cell proliferation and metastasis and indicates poor prognosis in breast cancer
Source: J Exp Clin Cancer Res. 2017 Jul 12;36:92. doi: 10.1186/s13046-017-0564-7 (PMC5506643; doi:10.1186/s13046-017-0564-7)

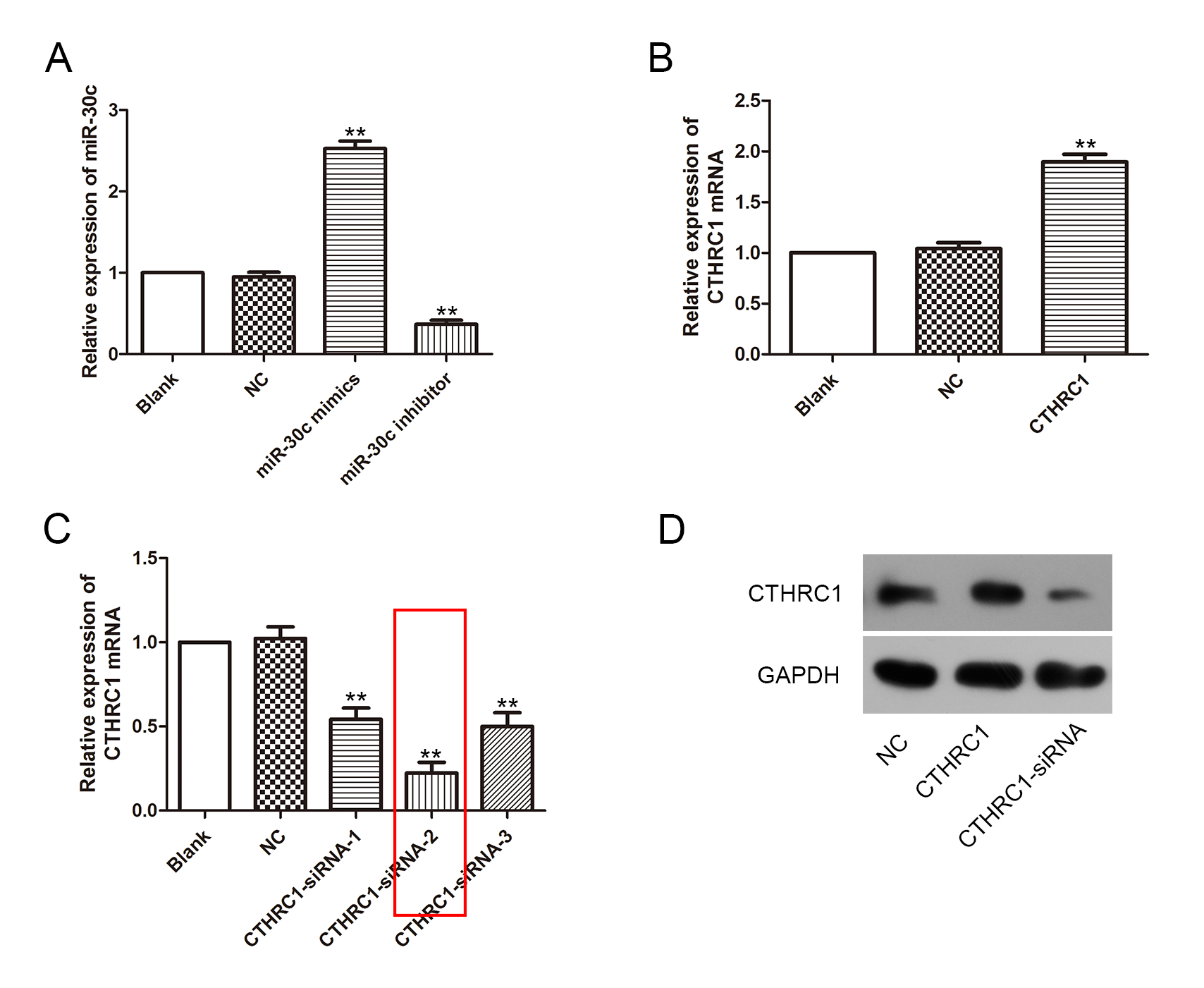

Supplement: Supplementary file 2 — To confirm the interference efficiency on miR-30c and CTHRC1. A, The relative expression level of miR-30c overexpression and inhibition in indicated cells was detected by qRT-PCR. **P < 0.01. B, The relative expression level of CTHRC1 overexpression in indicated cells was detected by qRT-PCR. **P < 0.01. C, Silencing efficiency of CTHRC1 in mRNA level by siRNA-1, −2 and −3 in indicated cells were identified by qRT-PCR. **P < 0.01. D, Overexpression and silencing efficiency of CTHRC1 in protein level were identified by western blot. (TIFF 503 kb) [file 13046_2017_564_MOESM2_ESM.tif]

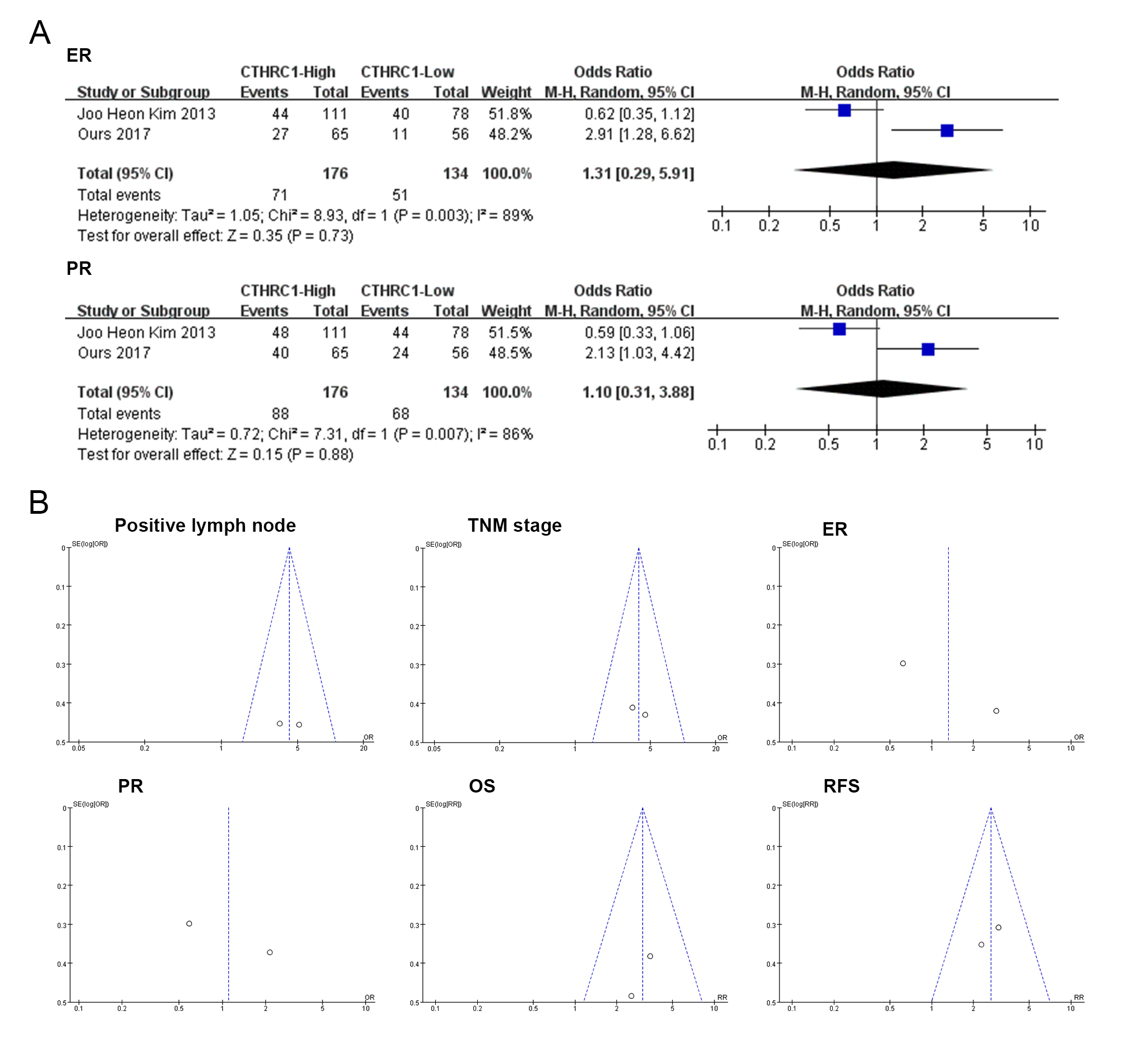

Supplement: Supplementary file 3 — Meta-analysis on prognostic value of CTHRC1. A, Forest plots showing the correlation of CTHRC1 with ER and PR. B, Funnel plots for publication bias. (TIFF 527 kb) [file 13046_2017_564_MOESM3_ESM.tif]

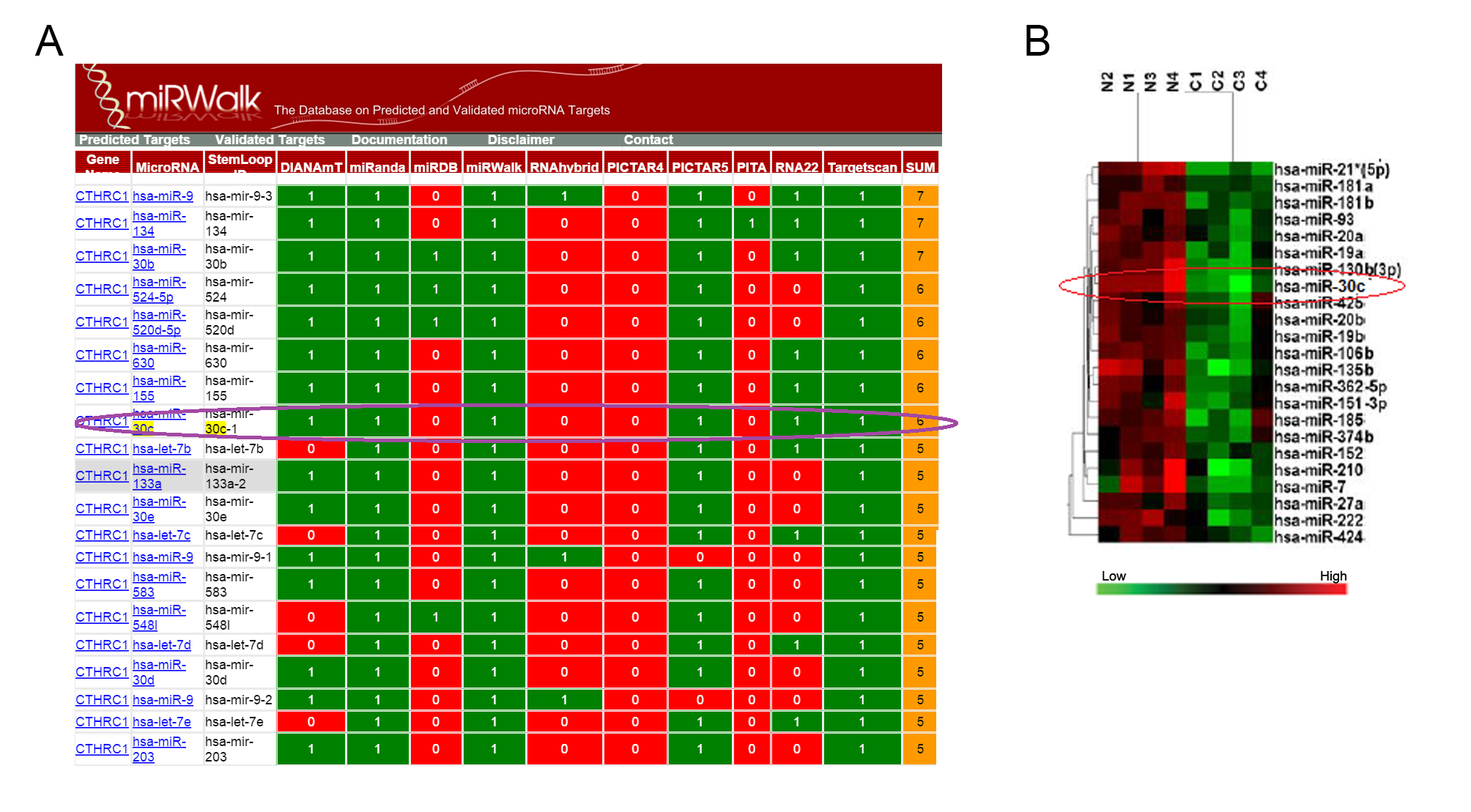

Supplement: Supplementary file 4 — To identify miRNAs that regulate CTHRC1 expression. A, miRwalk database identified potential miRNAs that bind to 3′ UTR of CTHRC1. B, miRNA microarray analysis revealed miR-30c was significantly down-regulated in breast cancer tissues. (TIFF 689 kb) [file 13046_2017_564_MOESM4_ESM.tif]
